# The Giant Protein Titin’s Role in Cardiomyopathy: Genetic, Transcriptional, and Post-translational Modifications of TTN and Their Contribution to Cardiac Disease

**DOI:** 10.3389/fphys.2019.01436

**Published:** 2019-11-28

**Authors:** Charles A. Tharp, Mary E. Haywood, Orfeo Sbaizero, Matthew R. G. Taylor, Luisa Mestroni

**Affiliations:** ^1^Adult Medical Genetics Program and Cardiovascular Institute, University of Colorado Anschutz Medical Campus, Aurora, CO, United States; ^2^Department of Engineering and Architecture, University of Trieste, Trieste, Italy

**Keywords:** titin, dilated cardiomyopathy, sarcomere, truncation variants, proportion-spliced-in, RBM20, phosphorylation, phosphosites

## Abstract

Dilated cardiomyopathy (DCM) is a leading cause of heart failure, sudden cardiac death and heart transplant. DCM is inherited in approximately 50% of cases, in which the most frequent genetic defects are truncation variants of the titin gene (*TTN*tv). *TTN* encodes titin, which is the largest protein in the body and is an essential component of the sarcomere. Titin serves as a biological spring, spanning half of the sarcomere and connecting the Z-disk to the M-line, with scaffold and signaling functions. Truncations of titin are believed to lead to either haploinsufficiency and loss-of-function, or to a “poison peptide” effect. However, other titin mechanisms are postulated to influence cardiac function including post-translational modifications, in particular changes in titin phosphorylation that alters the stiffness of the protein, and diversity of alternative splicing that generates different titin isoforms. In this article, we review the role of *TTN* mutations in development of DCM, how differential expression of titin isoforms relate to DCM pathophysiology, and discuss how post-translational modifications of titin can affect cardiomyocyte function. Current research efforts aim to elucidate the contribution of titin to myofibril assembly, stability, and signal transduction, and how mutant titin leads to cardiac dysfunction and human disease. Future research will need to translate this knowledge toward novel therapeutic approaches that can modulate titin transcriptional and post-translational defects to treat DCM and heart failure.

**HIGHLIGHTS:**

- Titin (TTN) truncation variants are the most frequent cause of dilated cardiomyopathy, one of the main causes of heart failure and heart transplant. Titin is a giant protein, and the mechanisms causing the disease are both complex and still incompletely understood.

- This review discusses the role of titin in myocardial function and in disease. In particular, we discuss TTN gene structure, the complexity of genotype-phenotype correlation in human disease, the physiology of TTN and the role of post-translation modification.

- Additional studies will be required to clarify whether missense variants are associated with cardiac disease. While initial studies suggested a role of non-synonymous variants in arrhythmogenic cardiomyopathy, confirmatory investigations have been hampered by the complexity of the protein structure and function.

## Introduction

**FIGURE 1:**
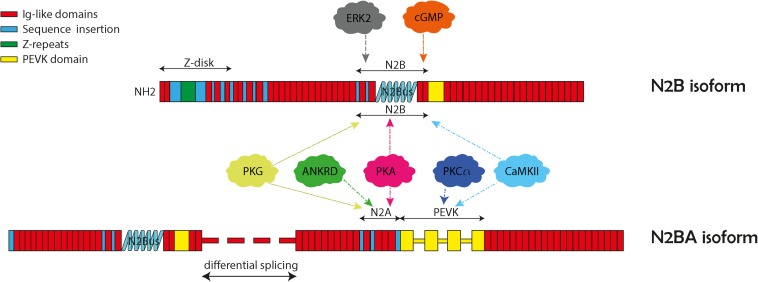
Structure and domains of titin N2B and N2BA isoforms. The two cardiac isoforms and their protein kinase phosphorylation N2B and N2BA are illustrated here with their specific protein regions. Both isoforms contain the N2B unique sequence (N2Bus), and the PEVK region (titin region rich in proline, glutamate, valine, and lysine). The N2BA isoform also contains the N2A unique sequence (N2Aus). The various protein kinase binding partners are indicated in the balloons with arrows pointing to their general binding regions.

The sarcomere is the basic structural unit that facilitates contraction of striated muscle. An essential component of the sarcomere is the giant filament protein titin (TTN). TTN is the largest protein in the human body, which is encoded by 364 exons of the *TTN* gene that produce a protein of between 27,000 and 33,000 amino acids in length with a molecular weight ranging between 2,900 and 3,800 kDa (Freiburg et al., 2000; LeWinter et al., 2007). Structurally, TTN serves as a biological spring, spanning half of the sarcomere and connecting the Z-disk to the M-line. It is composed of four structural subunits (Figure 1). The Z-line is the N-terminal region that embeds and anchors TTN to the sarcomere Z-disk. The I-band is composed of repetitive immunoglobulin (Ig) regions that can extend when mechanical force is applied, providing the extensible or “spring-like” function of TTN. The A-band is composed of Ig regions alternating with fibronectin and is a non-extensible rigid region that serves as a stable anchor for myosin binding during muscle contraction. The M-band is the C-terminal domain containing serine/threonine kinase domains and forms a scaffold with myomesin to link myosin to thick filaments at the M-line of the sarcomere. In addition to the essential structural role that TTN provides within the sarcomere it is also important for sarcomere formation, mechanosensing, and signal transduction.

Alternative splicing is a major feature of *TTN* and produces three major isoforms: N2A, N2B, and N2BA which predominately differ in the lengths of the extensible I-band domains. N2B and N2BA are isoforms expressed within adult cardiomyocytes and differ based on their length and extensibility, with N2BA being a longer, less rigid protein compared to N2B (Freiburg et al., 2000). *TTN* mutations are associated with cardiac diseases, particularly dilated cardiomyopathy (DCM) that presents with ventricular enlargement and systolic dysfunction in the absence of alternative etiologies of cardiomyopathy such as valvular, hypertensive, congenital, or ischemic causes (Bozkurt et al., 2016). DCM has an estimated prevalence of 1:250–1:500. DCM accounts for up to one half of heart failure cases (Hershberger et al., 2013; LeWinter and Granzier, 2013). With the advent of next generation sequencing, many gene mutations that cause DCM have been discovered with 50 genes causing genetic DCM (Hershberger et al., 2010, 2013; Pinto et al., 2016; McNally and Mestroni, 2017). Of the known genetic mutations that cause DCM, *TTN* mutations are the most common accounting for 20–25% of cases (LeWinter and Granzier, 2013). Specifically, TTNtv are highly associated with development of DCM (Herman et al., 2012; Merlo et al., 2013). In recent years, the mechanisms for how *TTN* and potential modifier genes contribute to cardiomyopathy have been more clearly elucidated. The purpose of this article is to review the role of *TTN* mutations in development of DCM. Specifically, we will discuss how location of TTNtv lead to DCM, review mechanisms for TTNtv leading to phenotypes, consider how differential expression of TTN isoforms relates to DCM pathophysiology, and discuss how post-translational modifications of TTN can affect cardiomyocyte function.

## Association of Ttn Truncation Position and Human Heart Disease

Although *TTNt*vs are associated with a large proportion of DCM, it is interesting that TTNtvs are also found in 2–3% of the general population who are asymptomatic (Golbus et al., 2012; Herman et al., 2012; Roberts et al., 2015). While some healthy TTNtv carriers may later develop DCM, the heterogeneity of pathogenicity of TTNtv has prompted efforts to better understand how mutational position influences phenotype. Roberts et al. completed *TTN* sequencing in a diverse cohort of >5,000 persons whose cardiac phenotypes were known (including healthy persons) to correlate truncation location position with phenotype. The data demonstrated that a key predictor of pathogenicity of a TTNtv is whether the affected exon is expressed in cardiac tissue (Roberts et al., 2015). TTNtv located in constitutive exons were more often pathogenic; whereas those located in exons that are minimally expressed in heart tissue or that can be rescued, or “bypassed,” by differential splicing had a lower risk of DCM. A score to predict whether a truncation mutation in TTN is pathogenic has been proposed as the PSI. The PSI calculates a percentage of TTN transcripts in which a given exon is spliced into an expressed transcript based on RNA sequencing data from human left-ventricular tissue. Therefore, a high PSI score suggests a high proportion of the total transcripts include a given exon in cardiac tissue. This study found that a TTNtv located in exons with a PSI >0.9 were associated with at 93% probability of pathogenicity (likelihood ratio-14) if discovered in a patient with a DCM phenotype (Roberts et al., 2015).

**FIGURE 2:**
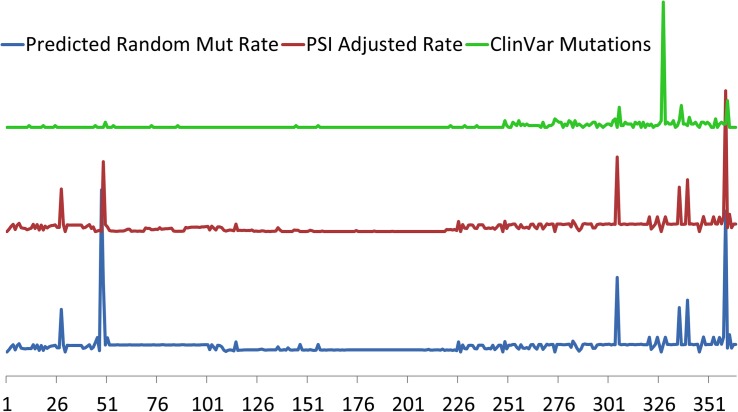
Relative predicted rates of purely random mutations in TTN based on exon size (bottom, blue) and adjusted for cardiac exon expression (middle red) using Proportion Spliced In (PSI) approach of Roberts et al. (2015). Regions of where PSI is generally <50% include exons 51–69, 78–89, and 124–220. Exon location of 327 clinically relevant TTN truncation variants from ClinVar (top, green) demonstrates clustering toward the carboxy terminus of TTN in the region of the A-band where most of the constitutively expressed exons are located. Exons indicated on horizontal axis.

Specific *TTN* variants may also be position-dependent with respect to proximal or distal ends of the protein with a correlation of pathogenicity to distance from N-terminal (Roberts et al., 2015; Schafer et al., 2017). This correlation of position to pathology however, is more likely a reflection of alternative splicing than true positional location, as TTNtv in DCM are overrepresented in the more distal A-band which has a higher proportion of constitutively expressed exons. In contrast, mutations in the more proximal exons are more likely to undergo alternative splicing (Roberts et al., 2015; Schafer et al., 2017). As an example, a large portion of TTNtv found in healthy controls are found in a single exon that represents an alternative 3′ exon that is exclusively expressed in the Novex-3 isoform which does not span the sarcomere and has very low expression in cardiomyocytes (Herman et al., 2012; Roberts et al., 2015; Schafer et al., 2017). The association of clinically relevant TTNtv with their expression is further demonstrated in Figure 2 where pathogenic mutations from the ClinVar database are clustered in the highly constitutively expressed A-band region located at the carboxy terminus.

## Mechanisms of Ttntv Cardiac Pathogenicity

The exact mechanisms for how TTNtv lead to the DCM phenotype have not been clearly demonstrated. In families with inherited TTNtvs, the heritability follows an autosomal dominant pattern, however, there is clearly incomplete and age-dependent penetrance, evidenced by patients who carry predicted pathogenic TTNtv who do not acquire the DCM phenotype, or do not develop DCM until later in life, respectively (Herman et al., 2012; Franaszczyk et al., 2017). The molecular mechanism for how TTNtv causes DCM has been controversial with several models proposed. It has been conjectured that TTNtv may lead to abnormally shortened TTN proteins that cause harm to sarcomere function and cardiomyocytes through a “poison peptide” mechanism. In order to support this model, identification of truncated TTN proteins and their association with sarcomere and cardiomyocyte dysfunction would be required, which has proven difficult. One model of the “poison peptide” has been suggested by the discovery of a specific missense mutation in *TTN* leading to a truncated protein that was discovered in two separate families with autosomal dominant inheritance of DCM. This CMD1G mutation was predicted to affect a highly conserved immunoglobulin fold at the Z-disc I-band transition (Gerull et al., 2002). In another example, induced pluripotent stem cell-derived cardiomyocytes (hiPS-CM) were made from a DCM patient with an A-band TTNtv. In this model, truncated TTN protein was isolated from the sarcomere and it was demonstrated that disorganization of the sarcomere led to reduction of contractile force by more than 50% (Hinson et al., 2015). Additionally, Chopra et al. described a direct mechanism by which TTNtvs affect sarcomere assembly related to loss of a binding site. Using hiPS-CM, it was shown that cells with heterozygous A-band TTNtv did not effectively form sarcomeres due to loss of binding site for β-cardiac myosin on TTN. This appeared to prevent formation of protocostameres and Z-disks leading to a reduction in diastolic tension (Chopra et al., 2018). These two studies support disease models by which TTNtv lead to abnormally shortened TTN protein and directly affect sarcomere function. Interestingly, changes in contractile force that were seen in hiPS-CM studies could not be recapitulated in a study of isolated cardiomyocytes derived from human hearts with TTNtvs (Vikhorev et al., 2017). This discrepancy in contractility between hiPS-CM and adult cardiomyocytes may be related to the relative developmental immaturity of hiPS-CM.

While there may be circumstances where some TTNtvs cause dominant negative effects on the sarcomere, an alternative model is haploinsufficiency. In this model, TTNtv leads to abnormal mRNA transcripts that are degraded by NMD (Zhou et al., 2015). From this, follows a reduction in allelic expression of TTN and this reduction could increase metabolic stress leading to compensatory changes in the cardiomyocyte and eventual phenotype of DCM (Tabish et al., 2017; Ware and Cook, 2018). Data supporting this model come from a study where A-band and I-band TTNtv in rats do not affect amount of TTN protein, however, increase NMD (Schafer et al., 2017). Furthermore it has been shown that TTNtv in A-band and I-band similarly affect cardiac metabolism by reducing medium and long chain fatty acids as nutritional substrates, instead of preferring branched-chain amino acids and glycolytic intermediates similar to metabolic changes seen in other models of heart failure (Shibayama et al., 2015; Schafer et al., 2017). It has been further proposed that changes in metabolism lead to activation of mTOR complex 1 signaling which causes pathogenic responses related to protein synthesis and autophagy (Ramos et al., 2012; Neishabouri et al., 2015; Schisler et al., 2015; Yano et al., 2016). Activation of the mTOR complex and associated pathways has been found in other forms of DCM suggesting they represent terminal maladaptive pathways (Yano et al., 2016; Sweet et al., 2018).

This model of haploinsufficiency leading to increased metabolic stress is also in keeping with the clinical presentation of DCM in patients with TTNtv who have incomplete penetrance and generally present later in life, and often in conjunction with additional cardiac stressors (Herman et al., 2012; Franaszczyk et al., 2017). In a recent prospective observational study of patients with DCM, patients with TTNtv presented at an advanced median age of 49, which is similar to other studies of TTNtv in DCM (Herman et al., 2012; Franaszczyk et al., 2017; Tayal et al., 2017). Compared to other etiologies of DCM, patients with TTNtvs had similar clinical prognosis with respect to a combined outcome of cardiovascular mortality, major arrhythmia, and major heart failure events (Tayal et al., 2017). This study also demonstrated no significant phenotype differences among TTNtvs in regards to mutation location suggesting TTNtvs similarly affected cardiac stress leading to DCM, arguing for a mutation independent haploinsufficiency model (Tayal et al., 2017). Interestingly, even without a phenotype of DCM, changes in LV size are seen in asymptomatic patients with TTNtv suggesting that TTNtv affect cardiac function in ostensibly healthy persons and that also there could be significant compensation prior to development of DCM (Schafer et al., 2017). Animal models similarly demonstrate this delayed or incomplete DCM phenotype. This is evidenced by a murine model described by Gramlich et al. (2015) where heterozygous TTNtv mutants did not develop DCM unless exposed to cardiac stress. This study also described a potential therapeutic target in treating TTNtvs by alternatively splicing out the mutant exon. It was demonstrated that heterozygous mice that developed DCM with cardiac stress could be rescued by antisense oligonucleotides that induce exon skipping of the mutant exon. By skipping the frameshift mutation containing exon, premature termination was avoided and a majority of the TTN protein was translated. In this model, the DCM phenotype was rescued as demonstrated by sarcomere formation and contractile performance (Gramlich et al., 2015). This study represents an intriguing proof of concept for therapy to treat patients with TTNtvs with antisense oligonucleotides.

## Ttn Transcriptional Isoform Switching Alters Passive Tension of the Sarcomere

Titin has many roles, including signal transduction and mechanosensing, which are reviewed elsewhere (Gautel, 2011; Tabish et al., 2017). In the context of DCM pathophysiology, as a large protein that bridges half of the sarcomere, TTN is essential for sarcomere stabilization and maintenance of passive and active tension. Within the I-band, Ig-domains serve as springs and provide elasticity to TTN and the sarcomere. Understandably, the role of TTN’s spring functions in diastolic relaxation is of interest. When the sarcomere is not stretched, the Ig-domains are relaxed and highly folded. When tension is applied, the domains extend to provide passive tension. Initial tensile forces stretch the distal Ig-domains followed by stretching of the PEVK segment with the N2B element providing the highest level of passive resistance (Trombitas et al., 1998; Kontrogianni-Konstantopoulos et al., 2009; Tabish et al., 2017). These four unique domains provide increased passive force as TTN is more highly extended. This tension-force relationship contributes to the physiologic function described in the Frank-Starling curve where increased diastolic volume leads to increased contractility (Fukuda and Granzier, 2005; Tabish et al., 2017).

One of the most important ways passive resistance is modulated in cardiomyocytes is through alternative expression of TTN isoforms. In healthy hearts, the TTN N2B isoform is predominant and the TTN N2BA isoform is less expressed (Nagueh et al., 2004). N2B is a comparatively shorter protein with fewer exons expressed from the I-band, and has less elasticity as a result of less Ig-like domains compared to the N2BA. Due to its larger size and increased elasticity, N2BA has less passive stiffness compared to N2B. In DCM, there is reduced systolic function and enlargement of the ventricles, which may be partially explained by decreased passive tension leading to less diastolic forces and resultant dilation of the heart. Experimentally it has been shown that heart tissue extracted from patients with DCM exhibit less passive tension when compared to controls (Nagueh et al., 2004). More interestingly in the same study, patients with DCM also had an increased ratio of N2BA:N2B TTN isoforms suggesting that reduced passive force from TTN isoform switching is a feature of DCM. The N2BA:N2B isoform ratios also correlated with echocardiographic findings of increased end diastolic volume, increased end systolic volume, and decreased systolic ejection fraction (Nagueh et al., 2004).

It has been conjectured that switching to the longer N2BA isoform is initially physiologic and improves diastolic function; however, it is likely that as the myocardium continues to fail, systolic function worsens due to decreased passive tension and diastolic pressure (Makarenko et al., 2004).

While regulation of isoform switching in TTN is likely complex, there has clearly been an association with the splicing factor RNA binding motif protein RBM20 (Guo et al., 2012). RBM20 is a splicing factor specific to muscle cells that is involved in formation of the spliceosome, which regulates mRNA splicing. Notably, mutations in *RBM20* are associated with a clear DCM phenotype in humans (Brauch et al., 2009; Li et al., 2010; Refaat et al., 2012). In a rat model, knockout of *RBM20* causes DCM and these rats have increased ratio of N2BA:N2B isoforms suggesting that RBM20 regulates isoform expression of TTN (Guo et al., 2012). Interestingly, in the same RBM20 knockout rats, viral expression of *RBM20* decreased the N2BA:N2B ratio (Guo et al., 2012). This suggests that upregulation of RBM20 can favor production of the shorter, stiffer N2B isoform and possibly rescue a DCM phenotype. These results were recapitulated in iPSC derived cardiomyocytes from a patient with an RBM20 missense mutation. These cells demonstrated a reduction in the amount of N2B expression leading to reduction in active and passive force when studied in engineered heart muscle (Streckfuss-Bomeke et al., 2017). Based on these results, control of RBM20 to alter N2BA:N2B may represent a potential therapeutic target for treating patients afflicted with DCM. Several cell signaling pathways have been implicated in regulation of RBM20 which subsequently leads to altered N2BA:N2B ratios. Recently, in neonatal rat cardiomyocytes, insulin was shown to activate the mTOR kinase axis leading to RBM20 dependent increase in N2B isoform expression (Zhu et al., 2017). Interestingly, the mTOR pathway is associated with development of DCM caused by TTNtv as described above. In addition, thyroid hormone, specifically T3, has been shown to upregulate N2B isoform expression via RBM20 in neonatal rat cardiomyocytes (Zhu et al., 2015).

One proposed mechanism by which RBM20 affects TTN alternative splicing is via regulation of circular RNA (circRNA) (Khan et al., 2016). CircRNA is formed during transcription when the spliceosome covalently binds the 5′ and 3′ ends of an exon forming a stable RNA molecule (Memczak et al., 2013). It is thought that circRNAs are co-generated with mRNAs and their formation regulates gene expression by competing with mRNA transcription to decrease availability of linear mRNAs (Ashwal-Fluss et al., 2014). In their paper, Khan et al. (2016) used ribosomal depleted RNA from human hearts and demonstrated significant circRNA from *TTN*. Furthermore, they showed that RBM20 knockout mice lack the TTN circRNA (Ashwal-Fluss et al., 2014). This study suggests that RBM20 is essential for formation of TTN circRNA and that circRNA is a pathway by which RBM20 modulates expression of alternative isoforms of TTN.

Control of RBM20 and subsequent expression of TTN N2BA:N2B ratios is also important in the development of diastolic dysfunction. It is conjectured that decreasing the N2BA:N2B ratio results in stiffening of TTN and a more rigid sarcomere. This in turn may lead to impaired relaxation and a less compliant ventricle consistent with heart failure with preserved ejection fraction (HFpEF). This is seen in a small group of patients who required heart transplantation due to heart failure. The patients with failing hearts but normal systolic function suggestive of diastolic dysfunction had a lower N2BA:N2B ratio compared to patients with failing hearts and reduced systolic function (Borbely et al., 2009). Furthermore in a mouse model of diastolic dysfunction, inhibition of RBM20 and increase in N2BA:N2B TTN expression led to improvement of HFpEF resulting in normalization of function by echocardiogram and exercise tolerance (Bull et al., 2016). This suggests that control of TTN stiffness is an important therapeutic target for patients with systolic as well as diastolic dysfunction.

## Post-Translation Modification of Titin

In addition to transcriptional modifications, TTN function is also highly regulated by post-translational modifications. These alterations of TTN allow the cardiomyocyte and sarcomere to rapidly adjust to environmental changes within the heart. The most well described modification to TTN involves phosphorylation and de-phosphorylation at unique sites within the protein. Due its size, TTN may have the most phosphorylation sites of any protein. Hundreds of phosphorylation sites on TTN have been predicted based on proteomic analysis. These are listed on online databases: http://gygi.med.harvard.edu/phosphomouse/index.php (Huttlin et al., 2010), http://www.phosphosite.org/ (Hornbeck et al., 2015) or http://cpr1.sund.ku.dk/cgi-bin/PTM.pl (Lundby et al., 2012). While much effort has been made to understand how phosphorylation of TTN affects its function within the cardiomyocyte, only a few of these phosphosites have been associated with changes in the structure and function of TTN (Linke and Hamdani, 2014; Krysiak et al., 2018). The effect that phosphorylation has on the function of TTN depends on the unique structural domain that is being altered. Phosphorylation on TTN can be categorized based on which domain is being altered. Most of the biologically significant phosphosites that have been described within TTN are located in “spring-like” I-domain. This is likely due to the dynamic properties of this domain, where phosphorylation has the most significant effects on the passive and active tension of the sarcomere. Within the I-domain, there are two specific elements where differential phosphorylation has been shown to directly alter the length and tension of cardiomyocytes.

The PEVK element within the I-band is highly extensible and is characterized by richness in proline, glutamate, valine, and lysine (Freiburg et al., 2000). Phosphorylation of this element by the kinase PKCα in mouse and pig cardiomyocytes has been shown to increase cardiomyocyte passive tension by 20–30% (Hidalgo et al., 2009). Similarly, human cardiomyocytes treated with PKCα demonstrated increased passive stiffness compared to controls (Rain et al., 2014). It is hypothesized that addition of the positively charged phosphate in the negatively charged PEVK element increases electrostatic attraction making it less extensible and increasing passive force (Hamdani et al., 2017). In humans with failing hearts, it has been shown that there is increased PKC mediated phosphorylation of the PEVK element, which corresponds with increased passive tension (Hamdani et al., 2013). This study was done in patients with both hypertrophic and DCM who likely have very different diastolic pressures. It may be interesting to observe phosphorylation of the PEVK element in only DCM patients, where it may be hypothesized that there would be reduced phosphorylation leading to reduced passive tension and a dilated phenotype.

In addition to phosphorylation of the PEVK element, there are several phosphorylation sites within the cardiac specific N2B element. The N2B element is expressed on both cardiac TTN isoforms N2B and N2BA and is composed of three Ig domains as well as a unique sequence, named N2Bus that has many phosphosites (Linke et al., 1999). This element has been shown to have biologically significant phosphorylation events mediated by several different kinases including PKA (Yamasaki et al., 2002), PKG (Kruger et al., 2009), ERK2 (Raskin et al., 2012), and CaMKIIδ (Hamdani et al., 2013). Both *in vitro* and *in vivo* studies where the N2Bus element is phosphorylated demonstrate filament lengthening, reduction in TTN stiffness and reduction in passive tension of the sarcomere and cardiomyocyte (Hidalgo et al., 2009; Kruger et al., 2009; Perkin et al., 2015; Hamdani et al., 2017). While kinases that phosphorylate TTN at the N2Bus have been extensively studied, there has been less research on the effects of dephosphorylation via phosphatases. In a recent study, protein phosphatase 5 (PP5) was demonstrated to dephosphorylate TTN at the N2Bus element resulting in increased passive tension of cardiomyocytes. This study is complimentary to research in kinases that passive tension of TTN can be differentially regulated via phosphorylation and dephosphorylation of the N2Bus region. It is known that the N2Bus region is acidic and carries a positive charge. Therefore, addition of a positively charged phosphate in this element increases propulsion forces that lengthens the domain and decreases stiffness of TTN (Hamdani et al., 2017).

Post-translational modification of TTN via phosphorylation can alter its stiffness and passive tension so that cardiomyocytes can respond to physiologic changes. These alterations may also become pathologic in heart failure where alterations in TTN phosphorylation may lead to worsening of passive and active forces reducing the ability of the heart to function normally. It is likely that in addition to phosphorylation, TTN is modified by many other post-translational pathways. Research is ongoing to identify additional post-translational modifications of TTN and therapeutic targets. In one recent trial, it was suggested that in a mouse model of diastolic dysfunction, metformin improved HFpEF by increasing phosphorylation the N2B element via PKA. It was conjectured that phosphorylation increased TTN compliance and improved diastolic dysfunction as measured by echocardiogram, pressure volume analysis, and exercise tolerance (Slater et al., 2019).

## Conclusion

As the largest protein in the human body, the extent of TTN’s role in cardiac physiology and disease is not yet completely understood. We are still learning how TTN contributes to myofibril assembly, stability, and signal transduction, and how perturbations in these processes can lead to cardiac dysfunction and human disease. We have reviewed how modification of TTN at the genetic, transcriptional, and post-translational levels can affect cardiac function by altering the passive and active forces of the sarcomere and cardiomyocyte. Undoubtedly, future research will continue to elucidate TTN’s large role in cardiac physiology and how transcriptional and post-translational modifications may contribute to DCM as well as normal cardiac physiology. Alteration of TTN represents potential therapeutic targets for genetic and acquired cardiomyopathies.

## Author Contributions

CT wrote the manuscript with contributions of MH and OS. MT, LM, and OS contributed figures and grant support. MH, OS, MT, and LM provided critical insights and editing of the final manuscript.

## Conflict of Interest

The authors declare that the research was conducted in the absence of any commercial or financial relationships that could be construed as a potential conflict of interest.

## Abbreviations

NMD: Non-sense mRNA decay
PSI: proportion-spliced-in
TTN: titin
TTNtv: titin truncation variation.
